# The Assessment of Science: The Relative Merits of Post-Publication Review, the Impact Factor, and the Number of Citations

**DOI:** 10.1371/journal.pbio.1001675

**Published:** 2013-10-08

**Authors:** Adam Eyre-Walker, Nina Stoletzki

**Affiliations:** 1 School of Life Sciences, University of Sussex, Brighton, United Kingdom; 2 Hannover, Germany; University of California Davis, United States of America

## Abstract

The assessment of scientific publications is an integral part of the scientific process. Here we investigate three methods of assessing the merit of a scientific paper: subjective post-publication peer review, the number of citations gained by a paper, and the impact factor of the journal in which the article was published. We investigate these methods using two datasets in which subjective post-publication assessments of scientific publications have been made by experts. We find that there are moderate, but statistically significant, correlations between assessor scores, when two assessors have rated the same paper, and between assessor score and the number of citations a paper accrues. However, we show that assessor score depends strongly on the journal in which the paper is published, and that assessors tend to over-rate papers published in journals with high impact factors. If we control for this bias, we find that the correlation between assessor scores and between assessor score and the number of citations is weak, suggesting that scientists have little ability to judge either the intrinsic merit of a paper or its likely impact. We also show that the number of citations a paper receives is an extremely error-prone measure of scientific merit. Finally, we argue that the impact factor is likely to be a poor measure of merit, since it depends on subjective assessment. We conclude that the three measures of scientific merit considered here are poor; in particular subjective assessments are an error-prone, biased, and expensive method by which to assess merit. We argue that the impact factor may be the most satisfactory of the methods we have considered, since it is a form of pre-publication review. However, we emphasise that it is likely to be a very error-prone measure of merit that is qualitative, not quantitative.

## Abstract 2

Because both subjective post-publication review and the number of citations are highly error prone and biased measures of merit of scientific papers, journal-based metrics may be a better surrogate.

**Author summary**

Subjective assessments of the merit and likely impact of scientific publications are routinely made by scientists during their own research, and as part of promotion, appointment, and government committees. Using two large datasets in which scientists have made qualitative assessments of scientific merit, we show that scientists are poor at judging scientific merit and the likely impact of a paper, and that their judgment is strongly influenced by the journal in which the paper is published. We also demonstrate that the number of citations a paper accumulates is a poor measure of merit and we argue that although it is likely to be poor, the impact factor, of the journal in which a paper is published, may be the best measure of scientific merit currently available.

## Introduction

How should we assess the merit of a scientific publication? Is the judgment of a well-informed scientist better than the impact factor (IF) of the journal the paper is published in, or the number of citations that a paper receives? These are important questions that have a bearing upon both individual careers and university departments. They are also critical to governments. Several countries, including the United Kingdom, Canada, and Australia, attempt to assess the merit of the research being produced by scientists and universities and then allocate funds according to performance. In the United Kingdom, this process was known until recently as the Research Assessment Exercise (RAE) (www.rae.ac.uk); it has now been rebranded the Research Excellence Framework (REF) (www.ref.ac.uk). The RAE was first performed in 1986 and has been repeated six times at roughly 5-yearly intervals. Although, the detailed structure of these exercises has varied, they have all relied, to a large extent, on the subjective assessment of scientific publications by a panel of experts.

In a recent attempt to investigate how good scientists are at assessing the merit and impact of a scientific paper, Allen et al. [1] asked a panel of experts to rate 716 biomedical papers, which were the outcome of research funded, at least in part, by the Wellcome Trust (WT). They found that the level of agreement between experts was low, but that rater score was moderately correlated to the number of citations the paper had obtained 3 years after publication. However, they also found that the assessor score was more strongly correlated to the IF of the journal in which the paper was published than to the number of citations; it was therefore possible that the correlation between assessor scores, and between assessor scores and the number of citations was a consequence of assessors rating papers in high profile journals more highly, rather than an ability of assessors to judge the intrinsic merit or likely impact of a paper.

Subsequently, Wardle [2] has assessed the reliability of post-publication subjective assessments of scientific publications using the Faculty of 1000 (F1000) database. In the F1000 database, a panel of experts is encouraged to select and recommend the most important research papers from biology and medicine to subscribers of the database. Papers in the F1000 database are rated “recommended,” “must read,” or “exceptional.” He showed, amongst ecological papers, that selected papers were cited more often than non-selected papers, and that papers rated must read or exceptional garnered more citations than those rated recommended. However, the differences were small; the average numbers of citations for non-selected, recommended, and must read/exceptional were 21.6, 30.9, and 37.5, respectively. Furthermore, he noted that F1000 faculty had failed to recommend any of the 12 most heavily cited papers from the year 2005. Nevertheless there is a good correlation between rates of article citation and subjective assessments of research merit at an institutional level for some subjects, including most sciences [3].

The RAE and similar procedures are time consuming and expensive. The last RAE, conducted in 2008, cost the British government £12 million to perform [4], and universities an additional £47 million to prepare their submissions [5]. This has led to the suggestion that it might be better to measure the merit of science using bibliometric methods, either by rating the merit of a paper by the IF of the journal in which it is published, or directly through the number of citations a paper receives [6].

Here we investigate three methods of assessing the merit of a scientific publication: subjective post-publication peer review, the number of citations a paper accrues, and the IF. We do not attempt to define merit rigorously; it is simply the qualities in a paper that lead a scientist to rate a paper highly; it is likely that this largely depends upon the perceived importance of the paper. We also largely restrict our analysis to the assessment of merit rather than impact; for example, as we show below, the number of citations, which is a measure of impact, is a very poor measure of the underlying merit of the science, because the accumulation of citations is highly stochastic. We have considered the IF, rather than other measures of journal impact, of which there are many (see [7] for list of 39 measures), because it is simple and widely used.

## Results

### Datasets

**Figure 1:**
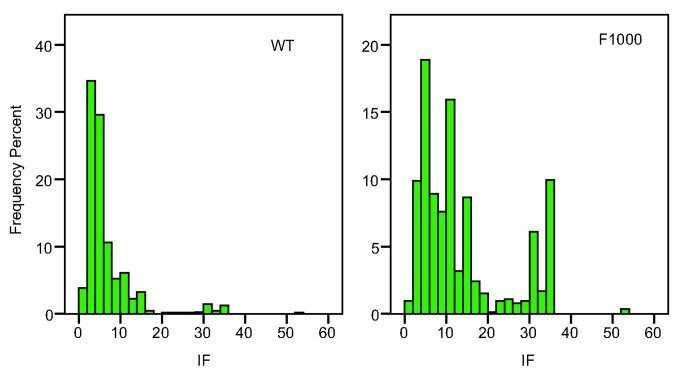
The distribution of the impact factor in the two datasets.

To investigate methods of assessing scientific merit we used two datasets [8] in which the merit of a scientific publication had been subjectively assessed by a panel of experts: (i) 716 papers from the WT dataset mentioned in the introduction, each of which had been scored by two assessors and which had been published in 2005, and (ii) 5,811 papers, also published in 2005, from the F1000 database, 1,328 of which had been assessed by more than one assessor. For each of these papers we collated citation information ∼6 years after publication. We also obtained the IF of the journal in which the paper had been published (further details in the Materials and Methods). The datasets have strengths and weaknesses. The F1000 dataset is considerably larger than the WT dataset, but it is papers that the assessors considered good enough to be featured in F1000; the papers therefore probably represent a narrower range of merit than in the WT dataset. Furthermore, the scores of two assessors are not independent in the F1000 dataset because the second assessor might have known the score of the first assessor, and F1000 scores have the potential to affect rates of citation, whereas the WT assessments were independent and confidential. The papers in both datasets are drawn from a diverse set of journals covering a broad range of IFs (Figure 1). Perhaps not surprisingly the F1000 data tend to be drawn from journals with higher IF, because they have been chosen by the assessors for inclusion in the F1000 database (Mean IF: WT = 6.6; F1000 = 13.9).

### Subjective Assessment of Merit

If scientists are good at assessing the merit of a scientific publication, and they agree on what merit is, then there should be a good level of agreement between assessors. Indeed assessors gave the same score in 47% and 50% of cases in the WT and F1000 datasets, respectively (Tables 1 and 2). However, we would have expected them to agree 40% of the time by chance alone in both datasets, so the excess agreement above these expectations is small. The correlations between assessor scores are correspondingly modest (WT r = 0.36, *p*<0.001; F1000 r = 0.26, *p*<0.001; all correlations presented in the text are summarized in Table S1—note Spearman's rank correlations are similar to Pearson's correlations for all analyses and these are given in Table S2). The correlation between assessor scores might be stronger in the WT dataset because the F1000 papers had been selected by the assessors as being good enough to rate; they therefore probably represent a narrower range of merit than in the WT data. Nevertheless the correlation in the F1000 dataset may have been inflated by the fact that the second assessor may have known the score of the first assessor.

**Table 1:** The correspondence between assessor scores for the WT dataset.

		Second Assessor			
		1	2	3	4
First assessor	1	60 (42)	97	13	0
	2	104	229 (222)	76	1
	3	12	59	42 (23)	8
	4	0	3	6	6 (0.3)

Table gives the number of papers rated 1 to 4 for the WT data. Figures in parentheses are the numbers expected by chance alone. Note the ordering of assessors is of no consequence in the WT data since the assessments were performed simultaneously and independently.

**Table 2:** The correspondence between assessor scores for the F1000 dataset.

		Second Assessor		
		Recommended	Must Read	Exceptional
First assessor	Recommended	365 (295)	197	39
	Must Read	240	255 (223)	76
	Exceptional	46	66	44 (19)

Table gives the number of papers rated recommended, must read, or exceptional for F1000 papers when both assessments were made within 12 months. Figures in parentheses are the numbers expected by chance alone. Note the second assessor scored the paper after the first assessor and may have known the score the first assessor gave.

**Figure 2:**
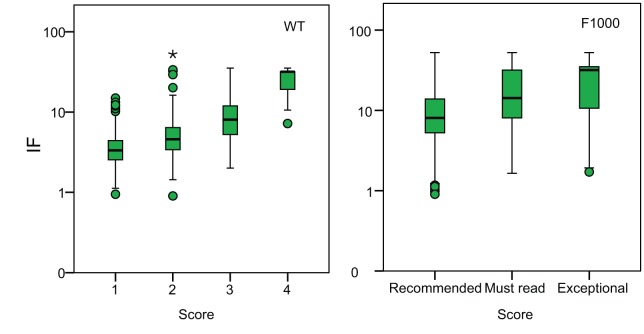
The correlation between assessor score and impact factor in the two datasets.

Strikingly, as Allen et al. [1] noted, there is a strong correlation between assessor score and the IF (WT r = 0.48, *p*<0.001; F1000 r = 0.35, *p*<0.001) (Figure 2); in fact the correlation is stronger than that between assessor scores. The correlation between assessor score and IF might arise for two non-mutually exclusive reasons. The correlation might be due to variation in merit and the ability of both assessors and journals to judge this merit; as a result, assessors might score better quality papers more highly and journals with high IFs might publish better quality papers. Alternatively, the correlation might be due to assessor bias; assessors might tend to rate papers in high IF journals more highly irrespective of their intrinsic merit. To investigate which of these explanations is correct, let us assume that the journal of publication does not affect the number of citations a paper accumulates; then the number of citations is likely to be a measure of merit. In fact, analyses of duplicate papers clearly show, as expected, that the journal affects the number of citations a paper receives, with papers in higher IF journals accumulating more citations for a given merit [9]–[11]; this makes our analysis conservative. Controlling the merit of a paper by using the number of citations as a measure of merit, we find a positive partial correlation between assessor score and IF (partial correlations: WT r = 0.35, *p*<0.001; F1000 r = 0.28, *p*<0.001). This suggests that assessors give higher scores to papers in high IF journals (or underrate the science in low IF journals), independent of their merit.

We can attempt to quantify the relative influence of IF and merit on assessor score by assuming that the number of citations is a measure of merit and then regressing assessor score against IF and the number of citations simultaneously; in essence this procedure asks how strong the relationship is between assessor score and IF when the number of citations is held constant, and between assessor score and the number of citations when IF is held constant. This analysis shows that assessor score is more strongly dependent upon the IF than the number of citations as judged by standardized regression gradients (WT, IF (b_s_ = 0.39) and citations (b_s_ = 0.16); F1000, IF (b_s_ = 0.30) and citations (b_s_ = 0.12)). The analysis underestimates the effect of the IF because the number of citations is affected by the IF of the journal in which the paper was published [9]–[11].

**Figure 3:**
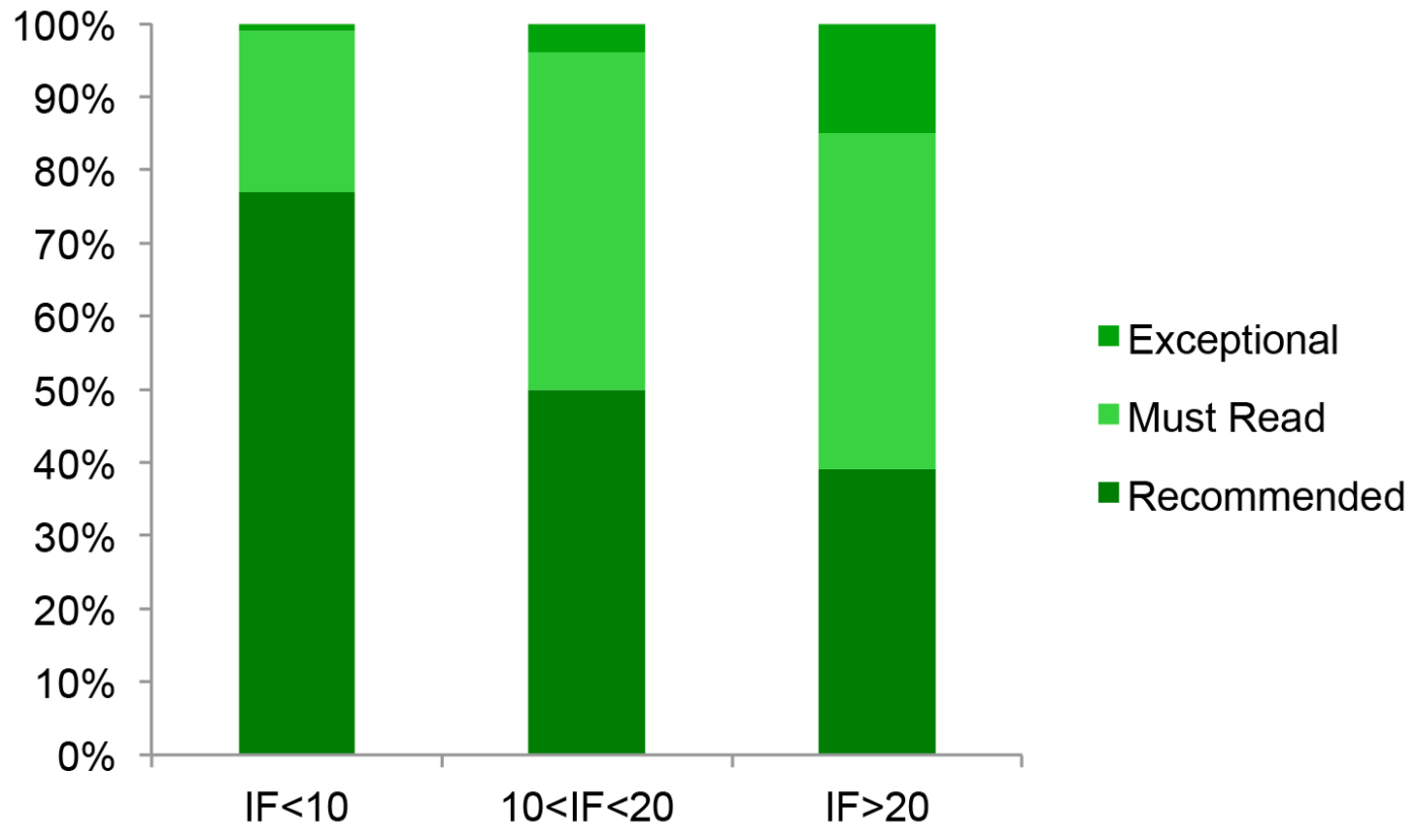
The proportion of papers, with between 90 and 110 citations in the F1000 dataset, scored in each category as a function of the IF of the journal in which the paper was published. The numbers of papers in each category are 131, 194, and 128 for IF<10, 10<IF<20, and IF>20, respectively.

The strength of the relationship between assessor score and the IF can be further illustrated by considering papers, in the largest of our datasets, that have similar numbers of citations to each other—those distributed around the mean in the F1000 dataset with between 90 and 110 citations (Figure 3). The proportion of papers scored in each of the three categories differs significantly across journals (chi-square test of independence *p*<0.001); the proportion that were rated either must read or exceptional is ∼2-fold higher in journals with IF>20 compared to those with IF<10 (*p*<0.001), and the proportion of papers rated exceptional is ∼10-fold higher (*p*<0.001).

If we remove the influence of IF upon assessor score, the correlations between assessor scores drop below 0.2 (partial correlations between assessor scores controlling for IF: WT, r = 0.15, *p*<0.001; F1000, r = 0.17, *p*<0.001). Similar patterns are observed within those journals in the F1000 dataset for which we have more than 100 papers; the correlations are typically very weak (Table 3) (average correlation between assessor scores within journals = 0.11, *p*<0.001).

**Table 3:** Correlations within journals with 100 or more papers in the F1000 dataset.

Journal	Correlation between Assessor Scores		Correlation between Assessor Score and the Number of Citations	
	*n* Papers	Correlation	*n* Papers	Correlation
Cell	114	0.23*	203	0.11
Current Biology	28	−0.16	103	0.23*
Development	22	−0.18	100	−0.089
Journal of Biological Chemistry	14	0.44	219	0.15*
Journal of Cell Biology	29	−0.022	103	0.22*
Journal of Neuroscience	12	−0.063	133	−0.057
Journal of the American Chemical Society	22	0.42	126	0.043
Molecular Cell	32	−0.049	121	0.15
Nature	217	0.15*	375	0.20***
Neuron	34	0.24	116	0.13
PNAS	115	0.32**	531	0.093*
Science	199	0.019	355	0.15**
				
Average		0.11		0.11

* *p*<0.05.

** *p*<0.01.

*** p<0.001.

We can quantify the performance of assessors as follows. Let us consider an additive model in which the score given by an assessor depends upon the merit of the paper plus some error. Under this model the correlation between assessor scores is expected to be 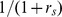 where 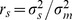, 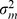 is the variance in merit and 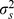 is the error variance associated with making an assessment (see Materials and Methods for derivation). If we assume that assessors are unaffected by the IF in making their assessment (which we have shown to be untrue) then we estimate, using the correlation between scores, that the error variance is approximately twice the variance in merit (WT 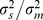 = 1.8 [bootstrap 95% confidence intervals of 1.4 and 2.5]; F1000 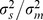 = 2.9 [2.2–3.9]). If we assume that the correlation between assessor score and IF is entirely due to bias, then we estimate, using the partial correlation between scores, controlling for IF, that the error variance is approximately 5-fold greater than the variance in merit within journals (WT 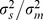 = 5.5 [3.3–13]; F1000 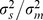 = 4.8 [3.4–7.7]). The true value lies somewhere between these extremes, however it is clear that an assessor's score is largely composed of error.

Overall it seems that subjective assessments of science are poor; they do not correlate strongly to each other and they appear to be strongly influenced by the journal in which the paper was published, with papers in high-ranking journals being afforded a higher score than their intrinsic merit warrants.

### Subjective Assessment of Impact

**Figure 4:**
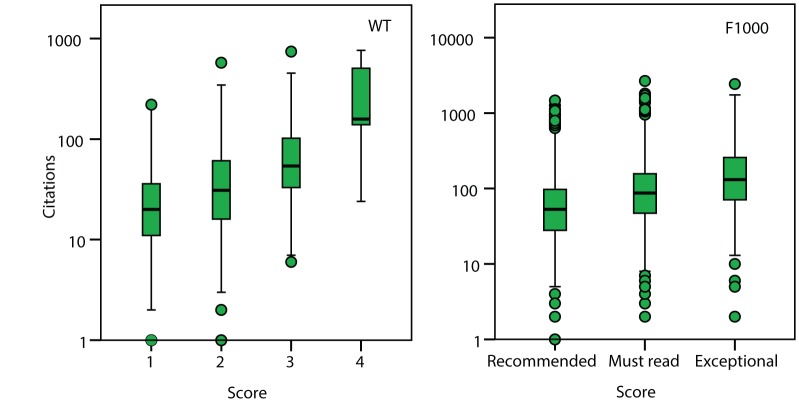
The correlation between assessor score and the number of citations in the two datasets.

Scientists appear to be poor at assessing the intrinsic merit of a publication, but are they better at predicting the future impact of a scientific paper? There are many means by which impact might be assessed; here we consider the simplest of these, the number of citations a paper has received. As with the correlation between assessor scores, the correlation between the assessor score and the number of citations a paper has accumulated are modest (WT r = 0.38, *p*<0.001; F1000 r = 0.25, *p*<0.001; the distribution of the number of citations is skewed but correlations using the log of the number of citations are similar to those for untransformed values [Table S3]) (Figure 4).

**Figure 5:**
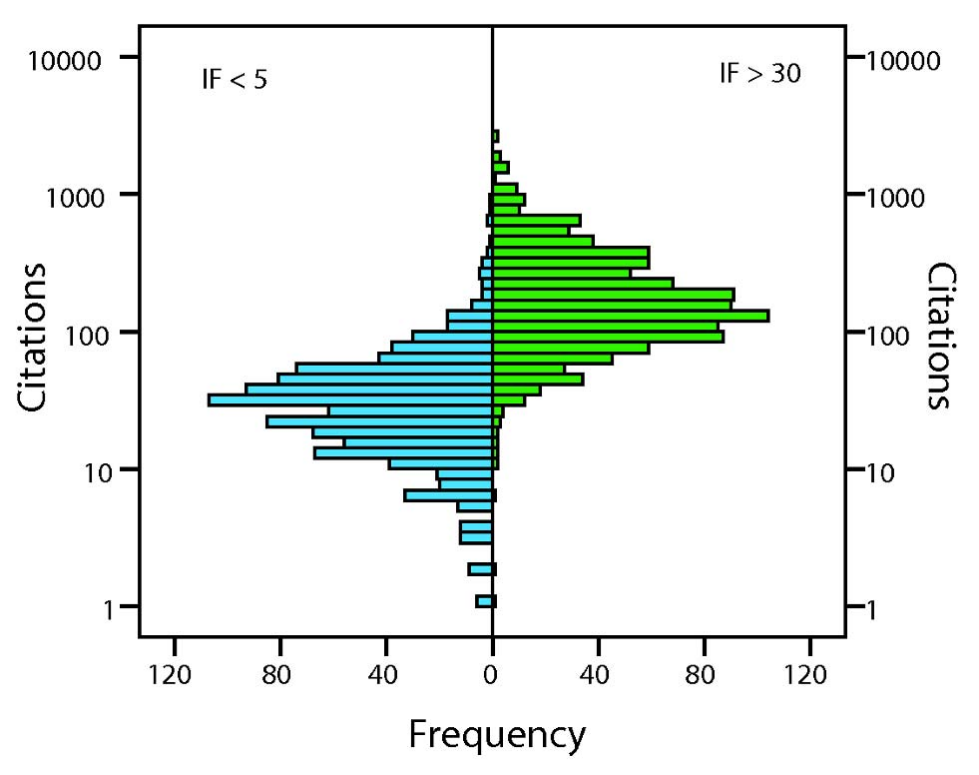
The distribution of the number of citations in journals with IF<5 and IF>30 in the F1000 dataset.

Part of the correlation between assessor scores and the number of citations may be due to the fact that assessors rank papers in high IF journals more highly (Figures 2 and 3) and by definition, papers in high IF journals are more highly cited. If we control for IF, we find that the correlation between assessor score and the number of citations becomes weak (partial correlations between assessor score and citations WT r = 0.15, *p*<0.001; F1000 r = 0.11, *p*<0.001). Similar patterns are observed within journals, for which we have enough data in the F1000 dataset (Table 3). The weak correlation between assessor score and the number of citations, controlling for IF or journal, means that assessor score explains less than 5% of the variance in the number of citations after controlling for IF; however, it should be appreciated that this is in part because the accumulation of citations is a highly stochastic process (see below). The low correlation between assessor score and the number of citations, controlling for IF, is not due to the lack of variation in the number of citations within journals; in all datasets there is more variance in the number of citations within journals than between them (the ratio of the within to the between journal variance in the number of citations is 1.6 and 3.7 in the WT and F1000 datasets, respectively) (Figure 5). The low partial correlation does not appear to be due to differences between fields either; if we re-run the regression of assessor score against IF and the number of citations in the F1000 dataset, but control for the assessor, and hence field of study, we get similar estimates to the analysis in which assessor is not controlled for (F1000 assessor score versus IF (b_s_ = 0.37) and citations (b_s_ = 0.092)).

### Number of Citations

An alternative to the subjective assessment of scientific merit is the use of bibliometric measures such as the IF of the journal in which the paper is published or the number of citations the paper receives. The number of citations a paper accumulates is likely to be subject to random fluctuation—two papers of similar merit will not accrue the same number of citations even if they are published in similar journals. We can infer the relative error variance associated with this process as follows. Let us assume that the number of citations within a journal is due to the intrinsic merit of the paper plus some error. The correlation between assessor score and the number of citations is therefore expected to be 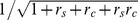 where 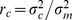 and 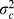 is the error variance associated with the accumulation of citations (see Materials and Methods for derivation). Hence we can estimate the error variance associated with the accumulation of citations relative to variance in merit by simultaneously considering the correlation between assessor scores and the correlation between assessor scores and the number of citations.

If we assume that assessors and the number of citations are unaffected by the IF of the journal, then we estimate the ratio of the error variance associated with citations to be approximately 1.5 times the variance in merit (WT *r_c_* = 1.5 [0.83–2.7]; F1000 *r_c_* = 1.6 [0.86–2.6]) and if we assume that the correlation between assessor score and IF is entirely due to bias then we estimate, using the partial correlation between score and citations, controlling for IF, that the ratio of the error variance to the variance in merit within journals to be greater than 5-fold (WT *r_c_* = 5.6 [1.2–42]; F1000 *r_c_* = 9.8 [4.0–31]). These estimates underestimate the error variance because they do not take into account the variance associated with which journal a paper gets published in; the stochasticity associated with this process will generate additional variance in the number of citations a paper accumulates if the journal affects the number of citations a paper receives, as analyses of duplicate papers suggest [9]–[11].

### Impact Factor

The IF might potentially be a better measure of merit than either a post-publication assessment or the number of citations, since several individuals are typically involved in a decision to publish, so the error variance associated with their combined assessment should be lower than that associated with the number of citations; although such benefits can be partially undermined by having a single individual determine whether a manuscript should be reviewed or by rejecting manuscripts if one review is unsupportive. Unfortunately, it seems likely that the IF will also be subject to considerable error. If we combine *n* independent assessments we expect the ratio of the error variance to the variance in merit in their combined qualitative assessment to be reduced by a factor *n*. Hence, if we assume that pre-publication assessments are of similar quality to post-publication assessments, and that three individuals have equal influence over the decision to publish a paper, their combined assessment is still likely to be dominated by error not merit; e.g., if we average the estimates of *r_s_* from the correlation between scores and between scores controlling for IF we have 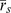 = 3.7 and 3.9, for the WT and F1000 datasets, respectively, which means that the ratio of the error variance associated with the combined assessor score will be ∼1.2× the variance in merit; i.e., the error variance is still larger than the variance in merit.

## Discussion

Our results have some important implications for the assessment of science. We have shown that scientists are poor at estimating the merit of a scientific publication; their assessments are error prone and biased by the journal in which the paper is published. In addition, subjective assessments are expensive and time-consuming. Scientists are also poor at predicting the future impact of a paper, as measured by the number of citations a paper accumulates. This appears to be due to two factors; scientists are not good at assessing merit and the accumulation of citations is a highly stochastic process, such that two papers of similar merit can accumulate very different numbers of citations just by chance.

The IF and the number of citations are also likely to be poor measures of merit, though they may be better measures of impact. The number of citations is a poor measure of merit for two reasons. First, the accumulation of citations is a highly stochastic process, so the number of citations is only poorly correlated to merit. It has previously been suggested that the error variance associated with the accumulation of citations is small based on the strong correlation between the number of citations in successive years [12], but such an analysis does not take into account the influence that citations have on subsequent levels of citation—the citations in successive years are not independent. Second, as others have shown, the number of citations is strongly affected by the journal in which the paper is published [9]–[11]. There are also additional problems associated with using the number of citations as a measure of merit since it is influenced by factors such as the geographic origin of the authors [13],[14], whether they are English speaking [14],[15], and the gender of the authors [16],[17] (though see [15]). The problems of using the number of citations as a measure of merit are also likely to affect other article level metrics such as downloads and social network activity.

The IF is likely to be poor because it is based on subjective assessment, although it does have the benefit of being a pre-publication assessment, and hence not influenced by the journal in which the paper has been published. In fact, given that the scientific community has already made an assessment of a paper's merit in deciding where it should be published, it seems odd to suggest that we could do better with post-publication assessment. Post-publication assessment cannot hope to be better than pre-publication assessment unless more individuals are involved in making the assessment, and even then it seems difficult to avoid the bias in favour of papers published in high-ranking journals that seems to pervade our assessments. However, the correlation between merit and IF is likely to be far from perfect. In fact the available evidence suggests there is little correlation between merit and IF, at least amongst low IF journals. The IF depends upon two factors, the merit of the papers being published by the journal and the effect that the journal has on the number of citations for a given level of merit. In the most extensive analysis of its kind, Lariviere and Gingras [11] analysed 4,532 cases in which the same paper had been published in two different journals; on average the two journals differed by 2.4-fold in their IFs and the papers differed 1.9-fold in the number of citations they had accumulated, suggesting that the higher IF journals in their analysis had gained their higher IF largely through positive feedback, not by publishing better papers. However, the mean IF of the journals in this study was less than one, and it seems unlikely that the IF is entirely a function of positive feedback amongst higher IF journals. Nevertheless the tendency for journals to affect the number of citations a paper receives means that IFs are NOT a quantitative measure of merit; a paper published in a journal with an IF of 30 is not on average six times better than one published in a journal with an IF of 5.

The IF has a number of additional benefits over subjective post-publication review and the number of citations as measures of merit. First, it is transparent. Second, it removes the difficult task of determining which papers should be selected for submission to an assessment exercise such as the RAE or REF; is it better to submit a paper in a high IF journal, a paper that has been highly cited, even if it appears in a low IF journal, or a paper that the submitter believes is their best work? Third, it is relatively cheap to implement. And fourth it is an instantaneous measure of merit.

The use of IF as a measure merit is unpopular with many scientists, a dissatisfaction that has recently found its voice in the San Francisco Declaration of Research Assessment (DORA) (http://am.ascb.org/dora/). The declaration urges institutions, funding bodies, and governments to avoid using journal level metrics, such as the IF, to assess the merit of scientific papers. Instead it promotes the use of subjective review and article level metrics. However, as we have shown, both subjective post-publication review and the number of citations, an example of an article level metric, are highly error prone measures of merit. Furthermore, the declaration fails to appreciate that journal level metrics are a form of pre-publication subjective review.

It has been argued that the IF is a poor measure of merit because the variation in the number of citations, accumulated by papers published in the same journal, is large [9],[18]; the IF is therefore unrepresentative of the number of citations that individual papers accumulate. However, as we have shown the accumulation of citations is highly stochastic, so we would expect a large variance in the number of citations even if the IF were a perfect measure of merit. There are however many problems with using the IF besides the error associated with the assessment. The IF is influenced by the type of papers that are published and with the way in which the IF is calculated [18],[19]. Furthermore it clearly needs to be standardized across fields. A possible solution to these problems may be to get leading scientists to rank the journals in their field, and to use these ranks as a measure of merit, rather than the IF. Finally, possibly the biggest problem with the IF is simply our reaction to it; we have a tendency to overrate papers published in high IF journals. So if are to use the IF, we need to reduce this tendency; one approach might be to rank all papers by their IF and assign scores by rank.

The REF will be performed in the United Kingdom next year in 2014. The assessment of publications forms the largest component of this exercise. This will be done by subjective post-publication review, with citation information being provided to some panels. However, as we have shown, both subjective review and the number of citations are very error prone measures of merit, so it seems likely that these assessments will also be extremely error prone, particularly given the volume of assessments that need to be made. For example, sub-panel 14 in the 2008 version of the RAE assessed ∼9,000 research outputs, each of which was assessed by two members of a 19 person panel; therefore each panel member assessed an average of just under 1,000 papers within a few months. We have also shown that assessors tend to overrate science in high IF journals, and although the REF [20], like the RAE before it [21], contains a stipulation that the journal of publication should not be taken into account in making an assessment, it is unclear whether this is possible.

In our research we have not been able to address another potential problem for a process such as the REF. It seems very likely that assessors will differ in their mean score—some assessors will tend to give higher scores than other assessors. This could potentially affect the overall score for a department, particularly if the department is small and its outputs scored by relatively few assessors.

The REF actually represents an unrivalled opportunity to investigate the assessment of scientific research and to assess the quality of the data produced by such an exercise. We would therefore encourage the REF to have all components of every submission assessed by two independent assessors and then investigate how strongly these are correlated and whether some assessors score more generously than others. Only then can we determine how reliable the data are.

In summary, we have shown that none of the measures of scientific merit that we have investigated are reliable. In particular subjective peer review is error prone, biased, and expensive; we must therefore question whether using peer review in exercises such as the RAE and the REF is worth the huge amount of resources spent on them. Ultimately the only way to obtain (a largely) unbiased estimate of merit is to have pre-publication assessment, by several independent assessors, of manuscripts devoid of author's names and addresses. Nevertheless this will be a noisy estimate of merit unless we are prepared to engage many reviewers for each paper.

## Materials and Methods

We compiled subjective assessments from two sources. The largest of these datasets was from the F1000 database (www.F1000.com). In the F1000 database a panel of experts selects and recommends papers from biology and medicine to subscribers of the database. Papers in the F1000 database are rated “recommended” (numerical score 6), “must read” (8), or “exceptional” (10). We chose to take all papers that been published in a single year, 2005; this was judged to be sufficiently recent to reflect current trends and biases in publishing, but sufficiently long ago to allow substantial numbers of citations to have accumulated. We restricted our analysis to those papers that had been assessed within 12 months of publication to minimize the influence that subsequent discussion and citation might have on the assessment. This gave us a dataset of 5,811 papers, with 1,328 papers having been assessed by two or more assessors within 12 months. We chose to consider the 5-year IFs, since it was over a similar time-scale to the period over which we collected citations. However, in our dataset the 2-year and 5-year IFs are very highly correlated (r = 0.99). Citations were obtained from Google Scholar in 2011. We also analysed the WT data collected by Allen et al. [1]. This is a dataset of 716 biomedical papers, which were published in 2005, and assessed within 6 months by two assessors. Papers were given scores of 4, landmark; 3, major addition to knowledge; 2, useful step forward; and 1, for the record. The scores were sorted such that the higher score was usually allocated to the first assessor; this will affect the correlations by reducing the variance within the first (and second) assessor scores. As a consequence the scores were randomly re-allocated to the first and second assessor. Citations were collated from Google Scholar in 2011. As with the F1000 data we used 5 year IFs from 2010. Data have been deposited with Dryad [8].

Because most journals are poorly represented in each dataset we estimated the within and between journal variance in the number of citations as follows. We rounded the IF to the nearest integer then grouped journals according to the integer value. We then performed ANOVA on those groups for which we had ten or more publications.

Estimates of the error variance in assessment relative to variance in merit can be estimated as follows. Let us assume that the score (*s*) given by an assessor is linearly dependent upon the merit (*m*) and some error (*e_s_*): *s* = *m*+*e_s_*. Let the variance in merit be 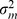 and that for the error be 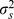, so the variance in the score is 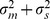. If two assessors score the same paper the covariance between their scores will simply be 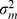 and the hence the correlation between scores is
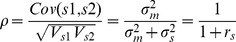
(1)where 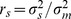.

If we similarly assume that the number of citations a paper accumulates depends linearly on the merit and some error (with variance 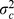) then the covariance between an assessor's score and the number of citations is 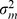 and the correlation is
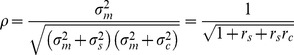
(2)where 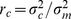. It is therefore straightforward to estimate *r_s_* and *r_c_*, and to obtain confidence intervals by bootstrapping the data.

## Supporting Information

Table S1 **The correlations, partial correlations, and standardized regression coefficients between assessor score (AS) and IF and the number of citations (CIT).** ***p<0.001.
(DOCX)

Table S2 **Spearman correlation coefficients between assessor scores and assessor scores and the number of citations and the IF.** ***p<0.001.
(DOCX)

Table S3 **The correlations, partial correlations, and standardized regression coefficients between assessor score (AS) and the log of IF and the log of the number of citations (CIT).** ***p<0.001.
(DOCX)

## Abbreviations

F1000: Faculty of 1000
IF: impact factor
RAE: Research Assessment Exercise
REF: Research Excellence Framework
WT: Wellcome Trust
