# Supplementary material for: The Assessment of Science: The Relative Merits of Post-Publication Review, the Impact Factor, and the Number of Citations
Source: PLoS Biol. 2013 Oct 8;11(10):e1001675. doi: 10.1371/journal.pbio.1001675 (PMC3792863; doi:10.1371/journal.pbio.1001675)
Supplement: Table S1 — The correlations, partial correlations, and standardized regression coefficients between assessor score (AS) and IF and the number of citations (CIT). ***p<0.001. (DOCX) [file pbio.1001675.s001.docx]

|  | Correlations and partial correlations | | | | | | Standardised slopes for regression of AS v. IF and Cit | |
| --- | --- | --- | --- | --- | --- | --- | --- | --- |
| Dataset | AS1 v AS2 | AS v Cit | AS v IF | AS v IF controlling Cit | AS1 v AS2 controlling IF | AS v Cit controlling IF | IF | Cit |
| WT | 0.36*** | 0.38*** | 0.48*** | 0.35*** | 0.15*** | 0.15*** | 0.39*** | 0.16*** |
| F1000 | 0.26*** | 0.25*** | 0.35*** | 0.28*** | 0.17*** | 0.11*** | 0.30*** | 0.12*** |

**Table S1.** The correlations and partial correlations between assessor score (AS) and IF and the number of citations (CIT). *** p<0.001
