# Supplementary material for: The Assessment of Science: The Relative Merits of Post-Publication Review, the Impact Factor, and the Number of Citations
Source: PLoS Biol. 2013 Oct 8;11(10):e1001675. doi: 10.1371/journal.pbio.1001675 (PMC3792863; doi:10.1371/journal.pbio.1001675)
Supplement: Table S2 — Spearman correlation coefficients between assessor scores and assessor scores and the number of citations and the IF. ***p<0.001. (DOCX) [file pbio.1001675.s002.docx]

| Dataset | Assessor score 1 v assessor score 2 | Assessor score 1 v no. of citations | Assessor Score 1 v IF |
| --- | --- | --- | --- |
| F1000 | 0.25*** | 0.28*** | 0.34*** |
| WT | 0.31*** | 0.37*** | 0.48*** |

**Table S2.** Spearman correlation coefficients between assessor scores and assessor scores and the number of citations and the IF. *** p<0.001
