# Supplementary material for: The Assessment of Science: The Relative Merits of Post-Publication Review, the Impact Factor, and the Number of Citations
Source: PLoS Biol. 2013 Oct 8;11(10):e1001675. doi: 10.1371/journal.pbio.1001675 (PMC3792863; doi:10.1371/journal.pbio.1001675)
Supplement: Table S3 — The correlations, partial correlations, and standardized regression coefficients between assessor score (AS) and the log of IF and the log of the number of citations (CIT). ***p<0.001. (DOCX) [file pbio.1001675.s003.docx]

|  | Correlations and partial correlations | | | | Standardised slopes for regression of AS v. Log(IF) and Log(Cit) | |
| --- | --- | --- | --- | --- | --- | --- |
| Dataset | AS v Log(Cit) | AS v Log(IF) | AS v Log(IF) controlling Log(Cit) | AS v Log(Cit) controlling Log(IF) | Log(IF) | Log(Cit) |
| F1000 | 0.29*** | 0.34*** | 0.22*** | 0.12*** | 0.26*** | 0.14*** |
| WT | 0.40*** | 0.52*** | 0.39*** | 0.16*** | 0.43*** | 0.16*** |

**Table S3.** The correlations and partial correlations between assessor score (AS) and the log of IF and the log of the number of citations (CIT). *** p<0.001
